# Circulating Regulatory T Cell Subsets in Patients with Sarcoidosis

**DOI:** 10.3390/diagnostics13081378

**Published:** 2023-04-10

**Authors:** Igor Kudryavtsev, Yulia Zinchenko, Anna Starshinova, Maria Serebriakova, Anna Malkova, Tatiana Akisheva, Dmitriy Kudlay, Anzhela Glushkova, Piotr Yablonskiy, Yehuda Shoenfeld

**Affiliations:** 1 Department of Immunology, Institution of Experimental Medicine, 197376 St. Petersburg, Russia; 2 Phthisiopulmonology Department, St. Petersburg Research Institute of Phthisiopulmonology, 194064 St. Petersburg, Russia; 3 Almazov National Medical Research Centre, 197341 St. Petersburg, Russia; 4 Laboratory of the Mosaic of Autoimmunity, St. Petersburg State University, 199034 St. Petersburg, Russia; 5 Department of Pharmacology, Sechenov First Moscow State Medical University, 119992 Moscow, Russia; 6 Institute of Immunology, 115552 Moscow, Russia; 7 Bekhterev National Research Medical Center for Psychiatry and Neurology, 19201 St. Petersburg, Russia; 8 Zabludowicz Center for Autoimmune Diseases, Sheba Medical Center, Tel Hashomer 5265601, Israel

**Keywords:** sarcoidosis, pathogenesis, autoimmunity, Treg, follicular Treg, Treg subsets

## Abstract

Over recent years, many researchers have supported the autoimmune theory of sarcoidosis. The presence of uncontrolled inflammatory response on local and system levels in patients with sarcoidosis did not define that the immunoregulatory mechanisms could be affected. The aim of this study was to evaluate the distribution and the disturbance circulating Treg cell subsets in the peripheral blood in patients with sarcoidosis. Materials and methods: A prospective comparative study was performed in 2016–2018 (34 patients with sarcoidosis (men (67.6%), women (32.3%)) were examined). Healthy subjects—the control group (*n* = 40). The diagnosis of pulmonary sarcoidosis was performed according to the standard criteria. We used two ten-color combinations of antibodies for Treg immunophenotyping. The first one contained CD39–FITC, CD127–PE, CCR4–PE/Dazzle™ 594, CD25–PC5.5, CD161–PC7, CD4–APC, CD8–APC–AF700, CD3–APC/Cy7, HLA–DR–PacBlue, and CD45 RA–BV 510™, while the second consisted of CXCR3–Alexa Fluor 488, CD25–РЕ, CXCR5–РЕ/Dazzle™ 594, CCR4–PerСP/Сy5.5, CCR6–РЕ/Cy7, CD4–АPC, CD8 АPC–AF700, CD3–АPC/Cy7, CCR7–BV 421, and CD45 RA–BV 510. The flow cytometry data were analyzed by using Kaluza software v2.3. A statistical analysis was performed with Statistica 7.0 and GraphPad Prism 8 software packages. Results of the study: Primarily, we found that patients with sarcoidosis had decreased absolute numbers of Treg cells in circulation. We noted that the level of CCR7-expressing Tregs decreased in patients with sarcoidosis vs. the control group (65.55% (60.08; 70.60) vs. 76.93% (69.59; 79.86) with *p* < 0.001). We noticed that the relative numbers of CD45RA–CCR7+ Tregs decreased in patients with sarcoidosis (27.11% vs. 35.43%, *p* < 0.001), while the frequency of CD45 RA–CCR7– and CD45RA+ CCR7– Tregs increased compared to the control group (33.3% vs. 22.73% and 0.76% vs. 0.51% with *p* < 0.001 and *p* = 0.028, respectively). CXCR3-expressing Treg cell subsets—Th1-like CCR60078CXCR3+ Tregs and Th17.1-like CCR6+ CXCR3+ Tregs—significantly increased in patients with sarcoidosis vs. the control group (14.4% vs. 10.5% with *p* < 0.01 and 27.9% vs. 22.8% with *p* < 0.01, respectively). Furthermore, the levels of peripheral blood EM Th17-like Tregs significantly decreased in the sarcoidosis group vs. the control group (36.38% vs. 46.70% with *p* < 0.001). Finally, we found that CXCR5 expression was increased in CM Tregs cell subsets in patients with sarcoidosis. Conclusions: Our data indicated a decrease in circulating Tregs absolute numbers and several alterations in Treg cell subsets. Moreover, our results highlight the presence of increased levels of CM CXCR5+ follicular Tregs in the periphery that could be linked with the imbalance of follicular Th cell subsets and alterations in B cell, based on the immune response. The balance between the two functionally distinct Treg cell populations—Th1-like and Th17-like Tregs—could be used in sarcoidosis diagnosis and the determination of prognosis and disease outcomes. Furthermore, we want to declare that analysis of Treg numbers of phenotypes could fully characterize their functional activity in peripherally inflamed tissues.

## 1. Introduction

Sarcoidosis is considered a granulomatous disease, subacute or chronic course, with lungs and mediastinal lymph nodes most often (up to 90% of cases) involved, as well as other organs and tissues, with granuloma formation without caseous necrosis [1]. There are two variants of the acute course of the disease: Löfgren’s Heerfordt-Waldenström syndrome [2]. The key feature of the pathogenesis of sarcoidosis is the formation of granulomas in the lungs, mediastinal lymph nodes, skin, and other organs. In patients who are genetically predisposed to this disease, a contact of antigen-presenting cells (macrophages, dendritic cells, epithelial cells) with unknown foreign antigen results in the dysregulated immune response that manifests in granulomatous inflammation [1,3]. The main characteristic of sarcoidosis is the formation of noncaseating epithelioid granulomas in various organs, represented by lymphocytes, epithelioid, and giant cells [2].

The problem of studying an etiological factor in research on sarcoidosis led to the identification of various infectious agents from bacteria to viral agents and fungi and inorganic factors (silicone, silicates, etc.) [3,4]. Therefore, there is an absence of a unified approach to therapy, as well as the possibility of conducting preclinical studies of the effectiveness treatment on sarcoidosis models [5].

Over recent years, many researchers have supported the autoimmune theory of sarcoidosis [1,6]. Etiology of the pulmonary sarcoidosis still remains unresolved; thus infections and/or autoimmunity are viewed as potential triggers of this disease [7].

Some studies demonstrated the presence of bacterial and fungal pathogens, or their absence, in the granuloma, so other etiologic mechanisms (autoimmunity with the presence of self-reactive T cells and auto-antibodies) were suggested as a possible cause of the disease [8,9,10]. The granuloma might be formatted with the inflammatory cascade, including the pro-inflammatory cytokines of T-helper (Th) cells, macrophages, and monocytes [1,11,12].

Recent studies have suggested that extended exposure of antigens with endotheliocytes, macrophages, and dendritic cells lead to differentiation of macrophages and secreting proinflammatory cytokines (TNF-α, IL-1). Antigens have presented the dendritic cells to T-lymphocytes, and they differentiated into CD4+, CD8+, Th17, and Treg, proliferated, and migrated to the focus of inflammation. Accumulation of epithelioid macrophages—B and T cells—in the focus of inflammation lead to the formation of epithelioid granulomas without foci of caseous necrosis [13]. Later, there is cellular damage by effectors of both humoral (antibodies) and the cellular immune response (cytokines of T-lymphocytes) [14,15].

Moreover, it was found that the presence of uncontrolled inflammatory responses on local and system levels in patients with sarcoidosis could impair the immunoregulatory mechanisms. Tregs play a key role in preventing autoimmune aggression. Tregs have an array of mechanisms of suppression that target a wide range of immune and non-immune cells (antigen-presenting cells, B cells, CD4+ and CD8+ T cells, and different effector cell in the site of inflammation), cell-to-cell contact-dependent suppression, production of anti-inflammatory cytokine (ectoenzymes CD39 and CD73), perforin/granzyme-mediated killing of target cells, etc. In sarcoidosis patients, Tregs were thought to be able to suppress granuloma development [16] and effector functions of different immune cells [17], although an investigation of the total peripheral blood Treg subset in sarcoidosis patients showed very contradictory data. Several studies showed an increase of Tregs in sarcoidosis patients compared with the control group [18,19]. Oppositely, several groups revealed that that the Treg level decreased during this disease [20,21]. Finally, it was shown that Tregs did not represent in peripheral blood samples obtained from sarcoidosis patients as well [22].

Another very important question is about the cell subset that can control self-reactive B cell activation, as well as self-reactive follicular Th (Tfh) activity in germinal center reaction. More recently, T follicular regulatory (Tfr) cells (a subset of T regulatory cells) were described in mice and humans [23]. It is thought that the Tfr cell plays the central part in the specific control of Tfh and B-cell interactions in the germinal center and displays suppressive capacities, preventing the emergence of a self-reactive clone of B cells. For example, mouse Tfr has phenotypic similarity with the surface profile of Tfh cells, including expression of cell surface CD4, CXCR5, PD-1, and ICOS, but also express characteristic markers for activated Tregs—CD25, CTLA-4, and GITR—and produce IL-10 in response to stimulation, as well as the main regulatory transcription factors for Tfh cells (Bcl6) and Tregs (Foxp3) [24]. Thus, the discovery of Tfr cells affected the biology humoral immunity but provided the target for therapy of B cell associated diseases. Currently, peripheral blood CXCR5-expressing Treg cells were studied in a very limited number of human diseases; in the first instance, they were studied in patients with autoimmune disorders, including ankylosing spondylitis [25], rheumatoid arthritis [26], multiple sclerosis [27], and many others diseases, including such viral infections as HIV [28], influenza [29] etc., but the biological importance of these cells in humans remains unclear so far.

The aim of this study was to evaluate the distribution and the disturbance circulating Treg cell subsets in the peripheral blood of patients with sarcoidosis.

## 2. Materials and Methods

### 2.1. Patient Characteristics

A prospective comparative study was performed in 2016–2018 in the St. Petersburg Scientific Research Institute of Phthisiopulmonology. A total of 34 patients with sarcoidosis stage 2 were examined. There were 23 men (67.6%) and 11 women (32.3%), with an average age of 32.7 ± 6.7 years. The clinical characteristics of the patients are in Table 1.

For performing the diagnosis of pulmonary sarcoidosis, we used the criteria of the American Thoracic Society (ATS), the European Respiratory Society (ERS), and the World Association of Sarcoidosis and Other Granulomatous Disorders (WASOG) [30]. The criteria included typical X-ray changes, epithelioid cell non-necrotizing granulomas, and the exclusion of other causes of granulomatous diseases, including tuberculosis.

Exclusion criteria were as follows: more than 2 years from X-ray changes, immunosuppressive and anti-tuberculosis therapy, plasmapheresis less than 2 months, HIV infection, syphilis, systemic autoimmunity, immunodeficiency, allergies and asthma, neoplastic diseases, and decompensated diabetes mellitus.

The inclusion criteria for the control group (*n* = 40) were as follows: no acute and chronic diseases and no tuberculosis infection, according to the immunological tests.

### 2.2. Methods of the Study

The examination of the patients included computed tomography (CT), blood tests, tests for tuberculosis infection (TB.T-SPOT, Mantoux test with 2 TE), and morphological examination of the lung and intrathoracic lymph node lesions (with transbronchial and videothoracoscopic biopsy).

### 2.3. Sample Collection

Peripheral blood samples (5 mL) were collected into BD Vacutainer^®^ blood collection tubes with K3-EDTA anti-coagulant (Becton, Dickinson and Company, Franklin Lakes, NJ, USA). Treg cell immunophenotyping was performed within ≤6 h after blood collection.

### 2.4. Regulatory T Cell Immunophenotyping

**Figure 1:**
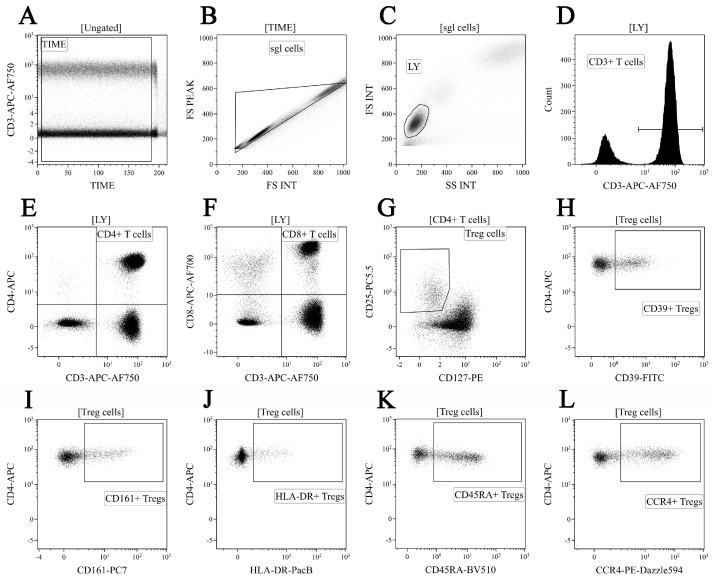
“Gating strategy” for main CD3+ T cell subsets and Treg cell surface functional antigens. Dot plot (**A**)—time gating; dot plot (**B**)—doublets exclusion; dot plot (**C**)—lymphocytes identification; dot plot (**D**)—total T cell subset gating; dot plot (**E**) and dot plot (F)—detection of CD4+ T cells and CD8+ T cells, respectively; dot plot (**G**)—Treg cells were gated as CD3 + CD4 + CD25 bright CD127 low-to-neg; dot plots (**H**–**L**)—examples of CD39, CD161, HLA-DR, CD45RA, and CCR4 to total Treg cell subsets, respectively.

Primarily, 200 μL of K3-EDTA-stabilized peripheral blood was incubated with the following fluorochrome-conjugated antibodies: CD39-FITC, CD127-PE, CCR4-PE/Dazzle™ 594, CD25-PC5.5, CD161-PC7, CD4-APC, CD8-APC-AF700, CD3-APC/Cy7, HLA-DR-PacBlue, and CD45 RA-BV 510™ (CD25, CD161, CD4, CD8, and HLA-DR were from Beckman Coulter, Pasadena, CA, USA and the other antibodies were manufactured by BioLegend, Inc., San Diego, CA, USA). The cells were stained according to the manufacturer’s manual (15 min at room temperature in the dark). Red blood cells were lysed for 15 min in the dark with 2 mL of VersaLyse Lysing Solution (Beckman Coulter, Brea, CA, USA), supplied with 50 μL IOTest 3 Fixative Solution (Beckman Coulter, USA). Finally, 200 μL of Flow-Count Fluorospheres (Beckman Coulter, USA) was used for absolute counting and was analyzed by flow cytometry using a 3/10 Navios flow cytometer (Beckman Coulter, Indianapolis, IN, USA). At least 40,000 CD4+ T cells were analyzed in each sample. Treg cells were gated as CD3+ CD4+ CD25 bright CD127 low-to-neg. The gating strategy is shown in Figure 1.

**Figure 2:**
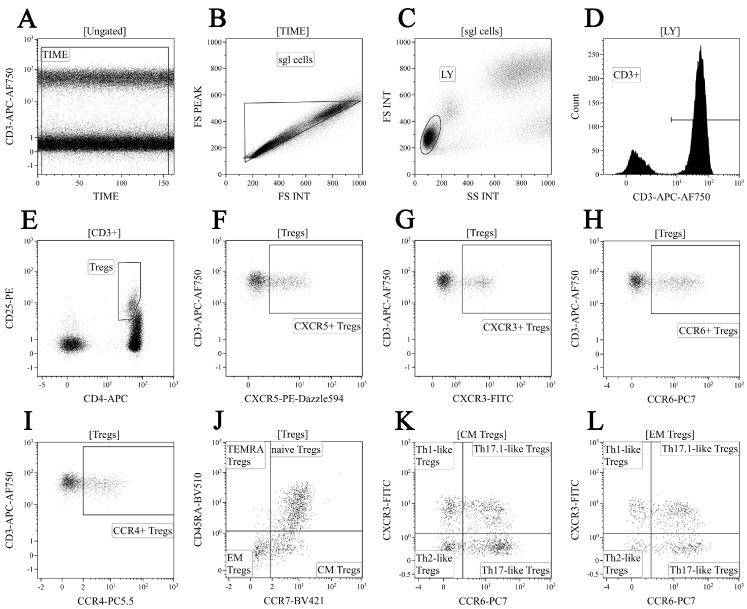
“Gating strategy” for ‘polarized’ Treg cell subset phenotyping. Dot plot (**A**)—time gating; dot plot (**B**)—doublets exclusion; dot plot (**C**)–lymphocytes identification; dot plot (**D**)—total T cell gating; dot plot (**E**)—regulatory T cell subsets were identified as CD3+ CD4+ CD25 bright within total CD3+ T cell subset; dot plots (**F**–**I**)—examples of CXCR5, CXCR3, CCR6, and CCR4 expression by Tregs, respectively; dot plot (**J**)—CD45RA and CCR7 co-expression were used to identify four main Treg maturation subsets, including CD45 RA+ CCR7+ ‘naïve’ (naïve Tregs), CD45 RA–CCR7+ central memory cells (CM Tregs), CD45 RA–CCR7 effector memory cells (EM Tregs), and CD45 RA+ CCR7 terminally-differentiated CD45 RA-positive effector memory Tregs (TEMRA Tregs); dot plots (**K**,**L**)—Th1-like Tregs were CCR6-CXCR3+, Th2-like Tregs were CCR6–CXCR3–, Th17-like Tregs were CCR6+ CXCR3–, and CCR6+ CXCR3+ “double-positive” Th17.1-like Tregs within central memory and effector memory Tregs, respectively.

We used a 10-color combination of fluorochrome-conjugated antibodies for ‘polarized’ Treg cell subset phenotyping, as it was described previously [31]. Peripheral blood samples were incubated with CXCR3-Alexa Fluor 488, CD25-РЕ, CXCR5-РЕ/Dazzle™ 594, CCR4-PerСP/Сy5.5, CCR6-РЕ/Cy7, CD4-АPC, CD8 АPC-AF700, CD3-АPC/Cy7, CCR7-BV 421, and CD45 RA-BV 510 (CD25, CD4, and CD8 were from Beckman Coulter, USA and other antibodies were manufactured by BioLegend, Inc., USA). The cell staining protocol was the same. Next, blood samples were washed twice with a sterile phosphate-buffered saline (PBS), supplied with 2% of heat inactivated fetal bovine serum (Sigma-Aldrich, St. Louis, MI, USA), and resuspended and fixed in 0.5 mL of fresh PBS, supplied with 2% neutral buffered formalin solution (Sigma-Aldrich, USA). Finally, all samples were analyzed by flow cytometry using 3/10 Navios flow cytometer (Beckman Coulter, USA). A total of 40,000 CD4+ T cells were collected in each sample. Treg cells were gated as CD3+ CD4+ CD25 bright. The gating strategy for the major ‘polarized’ Treg cell subsets is shown in Figure 2.

### 2.5. Statistical Analysis

Kaluza software v2.3 (Beckman Coulter, USA) was used for flow cytometry data analysis. CD3+ T cell subset concentrations were calculated by using Flow-Count Fluorospheres (Beckman Coulter, USA), according to the manufacturer’s manual. Statistical analysis was performed using GraphPad Prism 8 (Graph Pad software Inc., San Diego, CA, USA) and Statistica 7.0 (Stat Soft, Tulsa, OK, USA) software packages. Pearson’s chi-squared test was applied for testing normality of the data. All data were analyzed using non-parametric one-way analysis of variance (ANOVA) with Tukey’s multiple comparison test as a post-hoc test. Differences were considered statistically significant with a *p* < 0.05 value.

## 3. Results

### 3.1. Alterations in Circulating T Cell Subsets in Patients with Sarcoidosis

**Figure 3:**
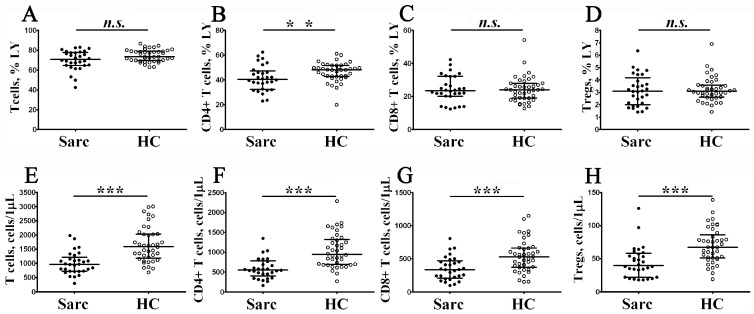
Alterations in main circulating T cell subsets from patients with sarcoidosis. Scatter plots (**A**–**D**) and (**E**–**H**) show the relative and absolute numbers of T cells, CD4+ T cells, CD8+ T cells, and Treg cells, respectively. Black circles—patients with sarcoidosis (Sarc, *n* = 34); white circles—the control group (HC, *n* = 40). Each dot represents an individual subject, and the data are shown as group medians and quartile ranges (Med (Q25; Q75)). The data were analyzed using non-parametric ANOVA with Tukey’s multiple comparison test as a post-hoc test **—*p* < 0.01; ***—*p* < 0.001).

To examine the absolute and relative numbers of main peripheral blood T cell subsets, including T cells, Th cells, CD8+ T cells, and regulatory T cells (Tregs), in patients with sarcoidosis, we analyzed CD3, CD4, CD8, CD25, and CD127 expression by flow cytometry (Figure 3).

Primarily, we noticed a decrease in the absolute number of all T cell subsets in sarcoidosis compared to the control group. We found that the absolute number of CD3+ T cells decreased in peripheral blood samples from patients with sarcoidosis vs. the control group (964 cells/1 μL (724; 1153) vs. 1595 cells/1 μL (1181; 2030) with *p* < 0.001). Similarly, we noticed that CD4+ T cells and CD8+ T cell concentrations were lower in patients with sarcoidosis (552 cells/1 μL (451; 763) vs. 945 cells/1 μL (691; 1321) with *p* < 0.001, and 335 cells/1 μL (222; 454) vs. 529 cells/1 μL (373; 664) with *p* < 0.001, respectively). Since we observed a decrease in CD4+ T cell levels, we also noticed a decrease of circulating Tregs in patients with sarcoidosis vs. in the control group (39 cells/1 μL (22; 57) vs. 67 cells/1 μL (51; 86) with *p* < 0.001). Next, we found that the relative numbers of CD4+ T cells decreased in patients with sarcoidosis vs. in the control group (40.56% (32.72; 46.24) vs. 48.18% (42.85; 51.70) with *p* < 0.01). We want to mention that lymphopenia is not uncommon in patients with sarcoidosis [32,33], and a reduced absolute number of total CD3+ T cells could affect the results measuring absolute numbers of all T cell subsets. Finally, we analyzed the ratio between Treg/Teff and found no differences in Tregs/CD4+ Teff ratio (0.072 (0.062; 0.089) vs. 0.069 (0.057; 0.075) with *p* > 0.05) and Tregs/CD8+ Teff ratio (0.137 (0.095; 0.191) vs. 0.133 (0.109; 0.188) with *p* > 0.05) between patients with sarcoidosis and the control group.

### 3.2. Altered Phenotype of Peripheral Blood Regulatory T Cells in Patients with Sarcoidosis

**Figure 4:**
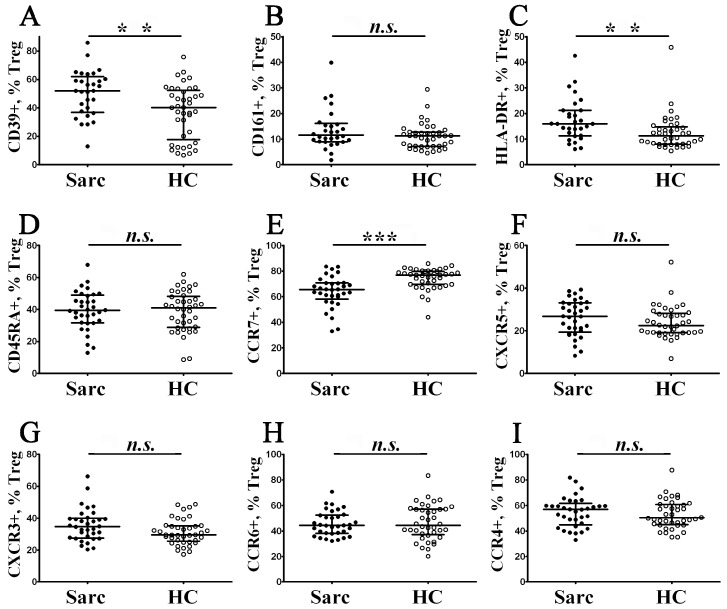
Alterations in Treg cell surface antigen expression in patients with sarcoidosis. Scatter plots (**A**–**I**) show the percentages of CD39+, CD161+, HLA–DR+, CD45 RA+, CCR7+, CXCR5+, CXCR3+, CCR6+, and CCR4+ cells within total Tregs, respectively. Black circles—patients with sarcoidosis (Sarc, *n* = 34); white circles—the control group (HC, *n* = 40). Each dot represents an individual subject, and the data are shown as group medians and quartile ranges (Med (Q25; Q75)). The data were analyzed using non-parametric ANOVA with Tukey’s multiple comparison test as a post-hoc test (**—*p* < 0.01; ***—*p* < 0.001).

Cell surface CD39 was identified as a functional Treg ectoenzyme, able to hydrolyze the proinflammatory ATP and the ADP to AMP; thereby, CD39 was expressed on effector/memory-like human Treg cells that displayed their anti-inflammatory activity through the formation of the extracellular immunosuppressive adenosine [34]. Moreover, we found that the level of CD39-expressing Tregs (Figure 4A) was increased in patients with sarcoidosis in comparison with the control group (53.03% (37.46; 60.22) vs. 40.16% (17.67; 52.30) with *p* < 0.01).

CD161 is a lectin-like receptor that was widely used as the marker of FoxP3+ Treg in humans that displayed inflammatory signatures, due to expression of proinflammatory cytokines, including IL-17 A, IFNγ, and IL-2 [35]. There was no difference in the amount of CD161-expressing Tregs (Figure 4B) between the sarcoidosis patients and the control group (11.54% (9.06; 15.72) vs. 11.24% (7.17; 12.67) with *p* > 0.05).

HLA-DR, also known as the cell surface molecule, which is characterized by the activation status of T cells, in the case of Tregs, allows us to identify a functionally distinct regulatory T cell subset involved in contact-dependent in vitro inhibition [36]. We found that HLA-DR-positive cell proportion (Figure 4C) within total Tregs was significantly higher in patients with sarcoidosis compared with the control group (15.94% (11.38; 21.20) vs. 11.26% (8.02; 14.85) with *p* < 0.01).

Previously, Miyara et al. found that, based on CD45RA expression, ‘naïve’ Tregs (CD45 RA+ FoxP3 lo) could be divided from effector-type Tregs (CD45 RA– FoxP3 hi) and cytokine-secreting CD45 RA–FoxP3 lo non-suppressive T cells [37]. We found no differences (Figure 4D) in the proportions of CD45 RA-expressing Tregs between the groups (39.40% (31.81; 48.53) in patients with sarcoidosis vs. 40.93% (28.90; 48.27) in the control group, *p* > 0.05).

Next, we examined the expression of five chemokine receptors which regulated Tregs directional migration into target organs. Primarily, we noted that the level of CCR7-expressing Tregs (Figure 4E) decreased in patients with sarcoidosis vs. the control group (65.55% (60.08; 70.60) vs. 76.93% (69.59; 79.86) with *p* < 0.001). It is known that CCR7 and its two ligands, CCL19 and CCL21, play an important part in the secondary lymphoid tissues homing of ‘naïve’ and regulatory T cells via high endothelial venules [38]. Moreover, we found no differences in CXCR5, CXCR3, CCR4, and CCR6 expression in cell membranes of Tregs between the groups (Figure 4F–I).

We have to mention that CXCR5 was necessary for peripheral blood follicular CD4+ T cells and B cells migration toward B cell follicles [39]. CXCR3 regulates the migration of different types of CD3+ T cells, to the sites of inflammation, along to CXCL9, CXCL10, and CXCL11 gradients [40]. CCR4 and its ligands, CCL17 and CCL22, are linked to skin homeostasis and inflammation [41]. Finally, CCR6 expression was critical for migration to inflamed mucosal tissues that enriched for CCL20 [42].

### 3.3. Identification of Phenotypically Distinct Maturation Subsets of Tregs in Sarcoidosis Patients

**Figure 5:**
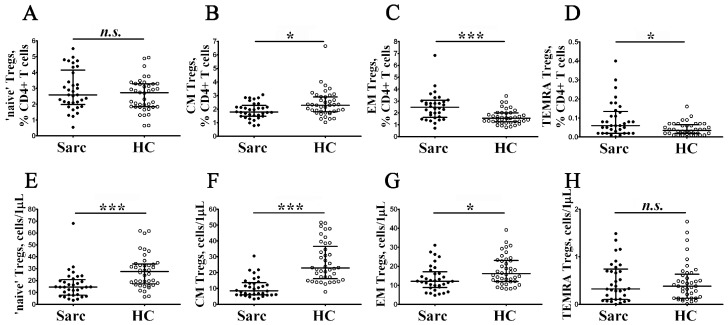
Alterations in relative and absolute frequencies of maturation Treg cell subsets in patients with sarcoidosis. Scatter plots (**A**–**D**) and (**E**–**H**) show the relative and absolute frequencies of ‘naïve’ (CD45 RA+ CCR7+), central memory (CM, CD45 RA–CCR7+), effector memory (EM, CD45 RA–CCR7–), and terminally differentiated CD45 RA-positive effector memory (TEMRA, CD45 RA+ CCR7–) Tregs, respectively. Black circles—patients with sarcoidosis (Sarc, *n* = 34); white circles—the control group (HC, *n* = 40). Each dot represents an individual subject, and the data are shown as group medians and quartile ranges (Med (Q25; Q75)). The data were analyzed using non-parametric ANOVA with Tukey’s multiple comparison test as a post-hoc test (*—*p* < 0.05; ***—*p* < 0.001).

Next, to determine whether there are differences in profiles of Treg subsets, we examined the expression of CD45RA and CCR7 on their cell membrane (Figure 2J). Accordingly, we divided Tregs into ‘naïve’ CD45 RA+ cells and memory CD45 RA–cells, the latter which were also subdivided into central memory CD45 RA–CCR7+ Tregs, effector memory CD45 RA–CCR7– Tregs (that were thought to be tissue-infiltrating Treg subsets), and CD45 RA+ CCR7– terminally differentiated Tregs [43,44]. We noticed that the absolute numbers of ‘naïve’, CM, and EM Tregs were significantly lower in patients with sarcoidosis compared to control group (Figure 5).

We found that the relative frequencies of CD45 RA–CCR7+ Tregs decreased in patients with sarcoidosis (1.79% (1.48; 2.15) vs. 2.29% (1.80; 2.91), *p* < 0.05), while the frequencies of CD45 RA–CCR7– and CD45 RA+ CCR7– Tregs increased in comparison with the control group (2.47% (1.68; 3.01) vs. 1.55% (1.26; 1.99) and 0.06% (0.02; 0.13) vs. 0.04% (0.02; 0.06) with *p* < 0.001 and *p* < 0.05, respectively).

### 3.4. Alterations of Chemokine Receptor Expression on Treg Maturation Subsets from Patients with Sarcoidosis

**Figure 6:**
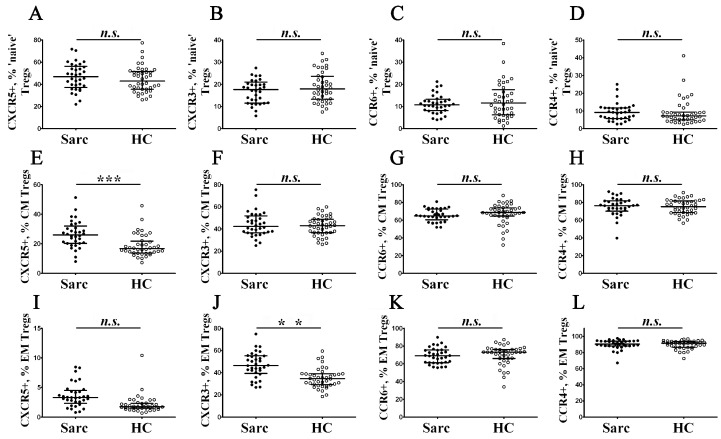
Chemokine receptor profiles of main maturation Treg cell subsets in patients with sarcoidosis. Scatter plots (**A**–**D**), (**E**–**H**), and (**I**–**L**) show the percentages of ‘naïve’, central memory, and effector memory Tregs, respectively, expressing CXCR5, CXCR3, CCR6, and CCR4, respectively. Black circles—patients with sarcoidosis (Sarc, *n* = 34); white circles—the control group (HC, *n* = 40). Each dot represents an individual subject, and the data are shown as group medians and quartile ranges (Med (Q25; Q75)). The data were analyzed using non-parametric ANOVA with Tukey’s multiple comparison test as a post-hoc test (**—*p* < 0.01; ***—*p* < 0.001).

Previously, we identified main Treg cell subsets according to different patterns of CD45RA and CCR7 expression, and then we decided to study phenotypic properties of ‘naïve’, CM, and EM Tregs (Figure 6).

We were interested in profiling chemokine receptors in TEMRA Tregs, but this subset was the smallest subpopulation of Tregs (accounting for less than 1% among Treg), and for this reason, further division to subsets, based on CXCR5, CXCR3, CCR6, and CCR4 expression, were not performed. Primarily, we found that CXCR5 expression increased in CM Treg cell subsets in patients with sarcoidosis (25.99% (20.34; 30.76) vs. 16.67% (13.73; 21.74) with *p* < 0.001). Next, we noticed that the relative number of CXCR3-expressing cells within EM Tregs were augmented in patients with sarcoidosis vs. the control group (46.47% (40.00; 55.01) vs. 34.53% (29.24; 38.97) with *p* < 0.01).

### 3.5. Imbalance in Main Peripheral Blood Treg Cell Subsets during Sarcoidosis

**Figure 7:**
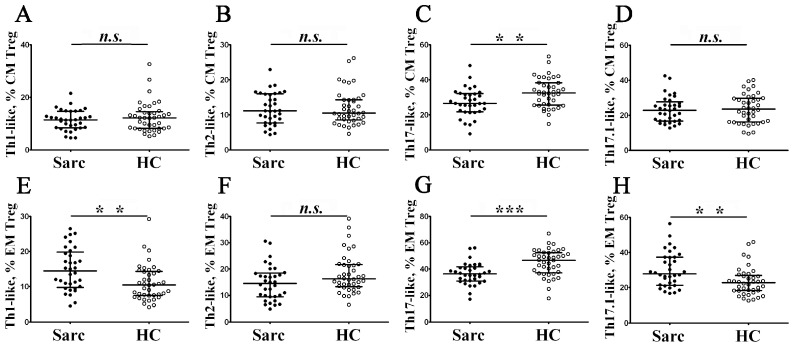
Imbalance of peripheral blood in Th1-like, Th2-like, Th17-like, and Th17.1-like cells in CM and EM Treg cell subsets in patients with sarcoidosis. Scatters plots (**A**–**D**) and (**E**–**H**) show the relative number of Th1-like (with CXCR5–CCR4+ CCR6–CXCR3+ phenotype), Th2-like (with CXCR5–CCR4+ CXCR3– phenotype), Th17-like (with CXCR5–CCR4+ CXCR3– phenotype), and Th17.1-like (with CXCR5-CCR4+ CCR6+ CXCR3+ phenotype) cells within central and effector memory Tregs, respectively. Each dot represents an individual subject, and the data are shown as group medians and quartile ranges (Med (Q25; Q75)). The data were analyzed using non-parametric ANOVA with Tukey’s multiple comparison test as a post-hoc test (**—*p* < 0.01; ***—*p* < 0.001).

Based on chemokine receptor CCR4, CCR6, and CXCR3 expression, we were able to identify distinct subsets of circulating Treg cells, localized with effector conventional Th cell subsets (Figure 2K,L are shown as the examples). Within CXCR5—a central and effector memory Treg cell—we identified CCR6–CXCR3+ Th1-like Tregs, CCR6–CXCR3– Th2-like Tregs, CCR6+ CXCR3– Th17-like Tregs, and CCR6+ CXCR3+ Th17.1-like Tregs, as it was suggested previously by Duhen et al. [45] and Halim et al [46]. In Figure 7, an imbalance of peripheral blood in Th1-like, Th2-like, Th17-like, and Th17.1-like cells in CM and EM Treg cell subsets in patients with sarcoidosis is presented.

We noticed that, within CD45 RA–CCR7+ central memory Tregs, the frequency of CCR4+ CCR6+ CXCR3– Th17-like Tregs decreased in patients with sarcoidosis compared to the control group (26.65% (21.98; 32.04) vs. 32.59% (25.73; 38.39) with *p* < 0.01). We analyzed Treg cell subsets in composition within CD45 RA–CCR7– effector memory Tregs that were able to migrate to peripheral inflamed tissues. We found that CXCR3-expressed sing Treg cell subsets—CCR4+ CCR6–CXCR3+ Th1-like and CCR4+ CCR6+ CXCR3+ Th17.1-like Tregs—were significantly increased in patients with sarcoidosis vs. in the control group (14.45% (10.00; 19.48) vs. 10.51% (7.50; 14.36) with *p* < 0.01 and 27.90% (21.54; 37.36) vs. 22.80% (18.32; 27.00) with *p* < 0.01, respectively). Furthermore, the levels of peripheral blood EM Th17-like Tregs significantly decreased in the sarcoidosis group vs. in the control group (36.38% (31.13; 40.99) vs. 46.70% (37.23; 52.54) with *p* < 0.001).

## 4. Discussion

Sarcoidosis is characterized by the epithelioid non-necrotizing granulomas with lymphocytes, epithelioid, and giant cells in various organs [7]. Macrophages, modified macrophages, epithelioid, and giant cells with CD4+ T cells are located in the central part of the granuloma, while CD8+ T cells, fibroblasts, macrophages, and fibrocytes are in the peripheral part of the granuloma. B lymphocytes are rarely presented in the granuloma [47]. Moreover, central necrosis might be detected [7]. It is important to note that the features of lymphocytic infiltration, and the general state of the immune system in sarcoidosis, is similar to some autoimmune diseases [48]. It was observed in the presence of autoimmune processes, impaired memory, and “naïve” B cell distribution [49] that there was an imbalance of T-helpers and T-follicular helpers towards an increase in the number of Th17, Tfh, Th2 and a decrease in Th1, T-regulatory cells [50], an imbalance between subpopulations of Tfh cells and regulatory Tfr [51,52], and elevated levels of short-lived, highly differentiated CD8+ T cells [53].

Defects in Treg cell numbers in circulation, alterations of their phenotype, and Tregs impaired functions in the site of inflammation, associated with the risk of autoimmune diseases in numerous models and human autoimmune diseases [54]. The data on Treg dynamics during sarcoidosis remains controversial in current literature. For instance, Huang et al. reported that the levels of Tregs from patients with sarcoidosis in bronchoalveolar lavage fluid (BALF) and peripheral blood samples decreased [21]. Similar data were obtained by Tondell et al. [55], Idali et al. [56], and Liu et al. [57]. We also noted that the absolute number of Treg cells decreased, while no differences were detected in relative numbers of Tregs within total lymphocyte population. Oppositely, Mroz et al. [58] and Oswald-Richter et al. [59] found increased numbers of Treg cells in blood samples and BALF in patients with active sarcoidosis. Furthermore, Broos et al. reported that increased levels of peripheral Treg cells were most considerable in patients with chronic disease, while the spontaneous resolution was not linked with increased levels of Tregs [60]. Interestingly, the frequency of Tregs increased in the relapsed patients compared with the stable patients [61].

It was suggested that sarcoidosis-associated lymphopenia was due to T-cell depletion [62,63]. Peripheral blood CD3+ lymphocytes could be depleted due to alterations of lymphogenesis (that could be linked with proinflammatory cytokine influences on T cell maturation in thymus), increased infiltration of target organs, or increased peripheral apoptosis induced by cell hyperactivation. The role of thymus and T cell thymus output is still not well understood in pathogenesis of sarcoidosis and lymphopenie, but the recent data indicated that thymus might play the critical role in pathogenesis of this disease in a certain group of patients, since the remission of skin and pulmonary sarcoidosis after thymectomy was noticed [64]. Furthermore, it was found that ‘naïve’ CD4+ T cells from patients with sarcoidosis were enriched for markers of non-TCR-mediated activation, apoptosis, and differentiation dysregulation [65]. Alterations in ‘naive’ CD4+ T cells activation could also be linked with low efficiency of Th cell maturation and ‘polarization’ in lymph nodes, which could result in low T cell levels in circulations. Finally, the high rates of proinflammatory factors in the sites of granuloma formation, that could be located in different tissues (especially in lungs), could also influence the level of CD3+ lymphocytes in circulation by recruiting both CD4+ and CD8+ T cells to the site of activation [17,66].

Thus, our analysis of circulating Treg frequency revealed no significant differences between sarcoidosis and the control group, but we found imbalance in Treg subsets expressing different patterns of CD45RA and CCR7. In particular, our investigation noted the normal level of Tregs that exhibited “naïve” phenotype, pointing to the absence of thymus dysfunctions, while the frequency of Treg subsets are thought to differentiate in peripheral lymph nodes. It was noted that Treg cells in sarcoidosis showed an increase of activated and memory Tregs and a decrease of resting and “naïve” Tregs [62]. Moreover, low levels of CM Tregs with hyperactivation enhanced CD95 expression [63]. An excessive proliferative response of Tregs, with increase frequencies of CD45 RO+ Ki67+ Tregs, were found in circulation [64], while the increased frequencies of CD45 RA–CCR7– Tregs in peripheral blood might be due to their impaired attraction or migration to sites of granuloma formation.

Indeed, we showed that sarcoidosis patient’s CD45 RA–CCR7– effector memory Tregs were able to migrate to peripheral inflamed tissues from the bloodstream, expressed high levels of CXCR3, and were enriched with Th1-like and Th17.1-like Tregs. It has been reported that alveolar macrophages from sarcoidosis patients secreted large amounts of CXCR3 ligands, including CXCL9, CXCL10, and CXCL11, that could play crucial roles in the accumulation of Tregs in the site of inflammation [65,66]. Similarly, the serum levels of CXCR3 ligands were also dramatically increased in sarcoidosis patients [67,68,69]. Furthermore, d’Alessandro et al. noted an increased expression in Tregs of tissue-homing cell surface molecule CD103 that stimulated cell migration to inflamed mucosal tissues [70]. All these data may point to the enhanced recruitment of Tregs into sarcoid lesions in different types of non-lymphoid tissues.

Next, we found increased levels of CXCR3-expressing Th1-like and Th17.1-like Treg cells in peripheral blood samples from patients with sarcoidosis, while CCR6-expressing Th17-like Tregs decreased. Interestingly, Th17- and Th1-like Treg cells phenotypically mimicked conventional Th17 and Th1 cells, but those IFN-γ- and IL-17-producing Treg cells also coproduced IL-10 in response to stimulation [46]. Furthermore, Th1-like Tregs expressed LAG3 and TIM-3 co-inhibitory receptors, as well as the cytolytic molecules GZMA and GZMK, while, by contrast, expression the TGF-β-activating molecule LRRC32 (GARP) was the highest in Th2-like Treg cells, and the decoy IL-1 receptor IL1 R2 was the most highly expressed by Th17-like and Th17.1-like Treg cells [71]. It was considered that those effector memory Treg cell subsets could migrate to peripheral inflamed tissues and were able to suppress their related effector Th cell counterparts in the site of inflammation [72].

Importantly, Th1-, Th17-, and Th17.1-like Tregs phenotypically mimicked conventional IFN-γ and IL-17-producting Th cells and expressed similar patterns of ‘homing’ molecules [46,73], including chemokine receptors that regulated Treg cell migration to the sites of inflammation [74,75]. It is known that circulating and BAL-associated CD4+ T cells that produce IFN-γ play an important role in sarcoidosis-associated inflammation and granuloma formation, just as serum and BAL from patients with sarcoidosis are characterized by the increased levels of IFN-γ [76,77,78]. Currently, accumulated results are now indicating that Th17.1 cells could play a central role in the pathogenesis of sarcoidosis. A significant increase was demonstrated in CCR6+ Th17.1 cells, which produced a large amount of IFN-γ in both BAL fluid and mediastinal lymph nodes in patients with sarcoidosis vs. healthy controls [13]. Furthermore, Broos et al. also noticed that the patients who did not resolve within these 2 years showed higher levels of IL-17 in their BAL compared to patients with sarcoidosis whose disease resolved. Similarly, Ramstein et al. showed that Th17.1 was markedly raised in BAL from sarcoidosis patients with progressive disease [79]. Interestingly, patients with Löfgren’s syndrome had increased levels of T-bet^+^ RORγt^+^ cells that produced IFN-γ and IL-17 A, co-expressed the chemokine receptors CXCR3 and CCR6, and their frequencies correlated with nonchronic disease [80].

It was noted that the imbalance of Th1-like and Th17-like Treg cell subsets could be linked with autoimmunity. For instance, Duhen et al. suggested that the increased frequency of IFN-γ-producing Treg cells in patients with type-1 diabetes and multiple sclerosis could be a response to autoimmune inflammation in these patients [45]. Next, E.L. McClymont et al. reported that FOXP3+ IFNγ+ Treg frequency was significantly increased in patients with type 1 diabetes compared to the control group [73]. Similarly, an effector memory Treg subset expressing HLA-DR, CCR4, CCR6, CXCR3, and GATA3 was increased in the high-risk group of patients with type 1 diabetes [74]. In patients with multiply sclerosis, the peripheral blood CCR6-expressing Treg decreased within total CD45 RA-Foxp3+ cell subset compared to healthy controls [75]. Controversially, IL-17-producing Treg cells were upregulated in patients with SLE, and the level of Th17-like cells in the peripheral blood was closely associated with the disease activity index (SLEDAI) [76]. An increased level of circulating Th17-like Tregs was noted in patients with idiopathic orbital inflammation, but Th17-like Tregs showed defective regulatory function since they failed to inhibit in vitro induced proliferation of ‘naïve’ T cells [77].

There are still a number of studies characterized by a regulatory subset of Tfh cells and T follicular regulatory (Tfr) cells in the context of human autoimmune diseases. Several studies on mouse models reported that Tfr cells could be derived from thymus derived Treg cells [78,79], but there is evidence suggesting that Tfr cells could be derived in the periphery from Foxp3– precursors in the context of a stimulus that promotes the conversion of conventional Foxp3– Th cells into Foxp3+ Treg cells [80]. Human Tregs are generated either during T cell antigen-independent differentiation in the thymus (tTregs) or during antigen-dependent differentiation in peripheral lymphoid organs from mature CD4 T cells in the periphery (pTregs), but the origin of human Tfh cell and their differentiation pathways are still known. Our data demonstrate that patients with sarcoidosis showed no differences with the control group in relative numbers of CXCR5+ cells within CD45 RA+ CCR7+ “naïve” or thymus-divided Tregs, while they had an increased number of CXCR5+ Tregs in the CD45 RA–CCR7+ subset. Thus, one can speculate that the imbalance in Tfr takes place in peripheral lymphoid tissues during a specific immune response to foreign or self-antigens. Recently, d’Alessandro et al. found potential evidence of the CD4 highCD25 highCXCR5 high cell’s role in sarcoidosis progression because patients showed higher percentages of Tfr cells in the peripheral blood vs. in the controls, and percentages of alveolar Tfr cells were positively correlated with Scadding stages [81]. Furthermore, alterations in follicular regulatory T cells could be linked with an imbalance in follicular Th cell subsets that were recently reported for patients with sarcoidosis [31,82].

Tfr was suggested to play an important part in autoimmunity, and recent studies have identified Tfr imbalance in several diseases. The significant increases of relative and absolute numbers of peripheral blood CD4+ CXCR5+ FoxP3+ Tfr cells in SLE patients compared with healthy controls were observed, as well as a positive correlation with SLEDAI score values [83]. The role of Tfr-like and Tfh-like was shown in patients with Sjögren syndrome, SS, myasthenia gravis, and ankylosing spondylitis [84,85,86,87]. Moreover, the correlation of Tfr-like/Tfh-like ratio with the clinical severity was revealed. In addition, there was an influence of glucocorticoid to the restoration of the imbalance of Tfr-like/Tfh-like cells, and, furthermore, the observed subset ratio restorations were also accompanied by lessened clinical symptoms.

## 5. Conclusions

Thus, alterations in Treg cell subsets and their phenotypes in patients with sarcoidosis could be attributed to dysregulated functions in the site of inflammation and granuloma formation. For a better understanding of the regulatory T cells’ contribution in sarcoidosis progression and Treg cell subsets we might identify their cell surface antigens as potential therapeutic targets for specific immune therapy in sarcoidosis. Our data highlight the presence of increased levels of CXCR5+ follicular Tregs in the periphery that could be linked with an imbalance of follicular Th cell subsets and alterations in B cell, based on the immune response. The balance between the two functionally distinct Treg cell populations—Th1-like and Th17-like Tregs—could be used in the future diagnoses of pulmonary sarcoidosis and the determination of prognosis and disease outcomes. A deeper characterization of Treg cells can lead to a better understanding of the pathogenetic mechanisms of sarcoidosis. Moreover, further investigation to specify the role of Treg cells and their distinct subsets in controlling inflammation in sarcoidosis pathogenesis will be necessary.

This study has several limitations. Primarily, in our first panel of monoclonal antibodies, we identified Treg cell as CD3+ CD4+ CD25 brightCD127 low-to-neg (Figure 1), while in the second panel of monoclonal antibodies, the Treg phenotype was CD3+ CD4+ CD25 bright (Figure 2), while FoxP3 expression for Treg determination was not used; this could be the limitation of our study. We also want to draw attention to the fact that the expression of CD25 was used to identify human Tregs on the total circulating CD4+ T cells for several decades [88], and it was used for Treg identification in many clinical and basic studies. Furthermore, bright expression of CD25 on human CD4+ T cells was also used to define peripheral blood Tregs, exhibiting the highest suppressive function [89]. Moreover, approximately 90% of CD25 high CD4+ T cells expressed FoxP3 [90,91]. Next, two independent groups of researchers found that a low or lack of expression of CD127 on the cell surface of CD25-expressing CD4+ T cells identified circulating peripheral blood Tregs with suppressive functions [92,93].

However, this CD25 vs. CD127 ‘gaiting strategy’ could not exactly correspond with CD4+ CD25+ FoxP3+ Tregs because approximately 15% of CD25+ CD127 low-to-neg cells did not express FoxP3 [92,93]; additionally, up to 10% of CD25+ FoxP3+ cells retained relatively high expression levels of CD127. However, it was also demonstrated that the CD4+ T cell of phenotype CD25 highCD127 low-to-neg with low-to-neg FoxP3 expression was equally immunosuppressive [94,95,96]. Moreover, almost all contemporary data on in vitro functional activity of Treg cells were received using cell-sorting of CD25 and/or CD127-expressing Tregs (current ‘strategies’ allow obtaining alive Treg cells for in vitro experiments), but not FoxP3-expressing Treg cells (intra nuclear FoxP3 staining requires cell fixation and permeabilization) [97,98]. On the other hand, there are FoxP3-negative Treg cell subsets—type 1 regulatory cells and IL-35-Producing T Cells—that did not express FoxP3 but effectively secreted the immunosuppressive cytokine IL-10 or IL-35, respectively [99] and [100], respectively. Another limitation is that our study was dedicated to the detailed analysis of the Tregs phenotype and to the identification of different Treg cell subsets, but we did not used in vitro functional tests. Furthermore, we want to mention that an analysis of Treg numbers of phenotypes could fully characterize their functional activity in peripheral inflamed tissues [101]. Finally, we used fresh peripheral blood samples for Treg cell analysis, but an absence of live/dead staining could be a limitation of our study.

Moreover, the sample size in our study was limited due to the availability and willingness of patients to donate large volumes of whole blood for the analyses, as well as to donate bronchoalveolar lavage. Furthermore, we did not use patients’ lung biopsy samples for immunophenotyping due to the small size of obtained material (lung biopsy samples were used only for diagnostic purposes but not for the research).

## Figures and Tables

**Table 1:** Clinical characteristics of patients with pulmonary sarcoidosis.

Characteristics:	Pulmonary Sarcoidosis, *n* (%), (*n* = 34)
Complaints:	
Clinical manifestations	28 (82.3)
Weakness	20 (58.8)
Cough	18 (52.9)
Dyspnea	10 (29.4)
Fever (37–37.9 °C)	10 (29.4)
Chest pain	3 (8.8)
Erithema nodosum	6 (17.6)
Arthralgia	7 (20.5)
Weight loss	5 (14.7)
X-ray findings:	
Enlarged lymph nodes	34 (100.0)
Foci in the lung tissue	31 (91.1)
Infiltation	3 (8.8)
Fibrosis	1 (2.9)
Ground-glass opacity	6 (17.6)
Medical history:	
Smoking	14 (41.1)
Family history of autoimmune diseases	4 (11.7)
Allergy	13 (38.2)
Results of TB testing:	
TB.T-SPOT test (positive)	0
Mantoux test with 2 TE (positive > 5 mm)	4 (11.7)

## Data Availability

All data were generated or analyzed during this study and are included in this published article.
